# Anesthetic management of super-elderly patients with remimazolam: a report of two cases

**DOI:** 10.1186/s40981-021-00474-4

**Published:** 2021-09-15

**Authors:** Junko Nakayama, Tomomi Ogihara, Rui Yajima, Yasushi Innami, Takashi Ouchi

**Affiliations:** 1 Department of Anesthesiology, Tokyo Dental College Ichikawa General Hospital, 5-11-13 Sugano, Ichikawa City, Chiba, 272-8513 Japan; 2 Department of Anesthesiology, Itakura Hospital, Chiba, Japan

**Keywords:** Remimazolam, Super-elderly, Bispectral index

## Abstract

**Background:**

Remimazolam is a newly developed benzodiazepine with more rapid onset and offset of sedation effects than midazolam. We report elderly patients in whom a small dose of remimazolam was successfully used for general anesthesia.

**Case presentation:**

Two elderly women (patients 1 and 2, aged 95 and 103 years, respectively) underwent hip fracture surgery under general anesthesia guided by bispectral index (BIS). Anesthesia was induced with 1.2 and 1.0 mg/kg/h and maintained with 0.2 and 0.1 mg/kg/h remimazolam, combined with fentanyl and remifentanil in patients 1 and 2, respectively. Their hemodynamics were stable with a small dose of vasopressor, and they awoke soon after the discontinuation of remimazolam without flumazenil reversal. Their postoperative courses were uneventful without any complications. Conversely, the remimazolam dose required to achieve adequate sedation were much lower than expected.

**Conclusion:**

Remimazolam could be useful in general anesthesia, particularly for super-elderly patients. However, the appropriate dose for induction and maintenance of anesthesia should be carefully considered based on BIS or vital signs.

## Background

Remimazolam, a new ultra-short-acting benzodiazepine, was approved for general anesthesia on January 23, 2020, in Japan for the first time in the world [1–3]. The effect of this agent rapidly appears and disappears compared with midazolam [4, 5]. Moreover, similar to other benzodiazepines, remimazolam agent can cause minimal cardiorespiratory depression [6–8]. These characteristics imply that remimazolam is particularly effective for short-term anesthesia in patients with poor performance status, including super-elderly patients. However, there is little real-world information on remimazolam under general anesthesia.

This report presents two cases of super-elderly women aged ≥ 95 years who underwent hip fracture surgery under general anesthesia using remimazolam guided by the bispectral index (BIS) or monitoring of the vital signs.

## Case presentation

### Case 1

A 95-year-old woman (135 cm and 40 kg) presented with right intertrochanteric femoral fracture requiring surgery. She had a history of hypertension, dyslipidemia, and dementia, which were controlled by amlodipine and olmesartan, pravastatin, and donepezil, respectively. Physical examination findings were normal, and laboratory examination revealed mild anemia with hemoglobin (Hb) level of 11.8 g/dL and coagulopathy of prothrombin time [PT] of 58% (PT-international normalized ratio, 1.3) probably owing to advanced age. Transthoracic echocardiography, electrocardiography, and respiratory function test revealed no abnormalities.

**Fig. 1:**
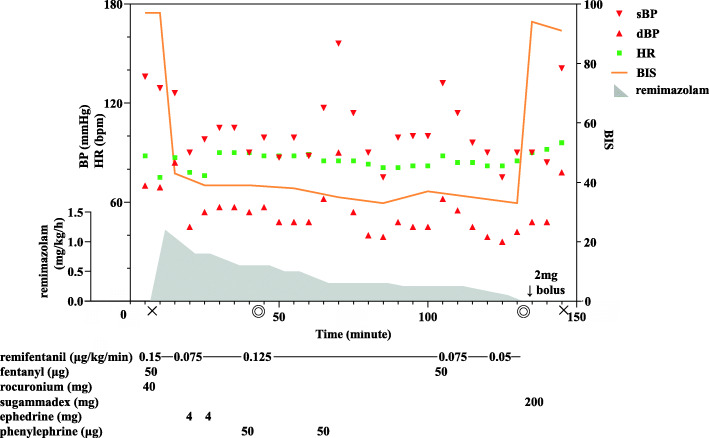
Anesthetic chart for case 1. Double circles and crosses represent the start/end of surgery and start/end of anesthesia, respectively. sBP, systolic blood pressure; dBP, diastolic blood pressure; HR, heart rate; BIS, bispectral index

Anesthetic chart is shown in Fig. 1. In the operating room, the patient’s vital signs were as follows: blood pressure (BP), 136/70 mmHg; heart rate, 88 bpm; and oxygen saturation on room air, 92%. BIS value was > 90 before inducing anesthesia. General anesthesia was induced by intravenous administration of fentanyl (50 μg), remifentanil (0.15 μg/kg/min), and remimazolam (1.2 mg/kg/h). She lost consciousness 4 min after initiation, and subsequently tracheal intubation was performed after administration of rocuronium (40 mg). After the operation was started, the remimazolam dose was adjusted so that the BIS value became 40–60 concomitantly with 0.125 μg/kg/min of remifentanil infusion. Eventually, remimazolam dose was decreased to 0.2 mg/kg/h although BIS value remained < 40. The operation was uneventfully completed with a small amount of blood loss in approximately 90 min. During the operation, she remained hemodynamically stable with administration of ephedrine 4 mg bolus and phenylephrine 50 μg bolus only twice to maintain systolic BP at > 90 mmHg, without the need of continuous administration of vasopressor agents. At the time of suture, 50 μg of fentanyl was administered, the remifentanil dose was decreased to 0.075 μg/kg/min, and remimazolam was maintained at 0.2 mg/kg/h. After completion of the operation, administration of these agents was discontinued and 200 mg of sugammadex was administered. Although 2.0 mg of remimazolam was administered as a bolus owing to the BIS value of > 70 during postural change for radiographic examination, 10 min after the bolus administration, she recovered consciousness without flumazenil and received extubation. Any consciousness problem, including resedation or postoperative cognitive function disorder, was not observed during the postoperative course. After rehabilitation, she was discharged on postoperative day 15.

### Case 2

A 103-year-old woman (approximately 150 cm and 40 kg) presented with right intertrochanteric femoral fracture requiring surgery. She had a history of chronic kidney disease (CKD) due to hypertension, which was treated with amlodipine, furosemide, and valsartan. Moreover, she took ticlopidine for the prevention of cerebral infarction. Physical examination results were normal, and laboratory examination revealed stage 3b CKD with serum creatinine level of 1.18 mg/dL (estimated glomerular filtration rate, 31.6 mL/min/1.73 m^2^) and mild anemia with Hb level of 10.0 g/dL. Transthoracic echocardiography and electrocardiography revealed no abnormalities.

**Fig. 2:**
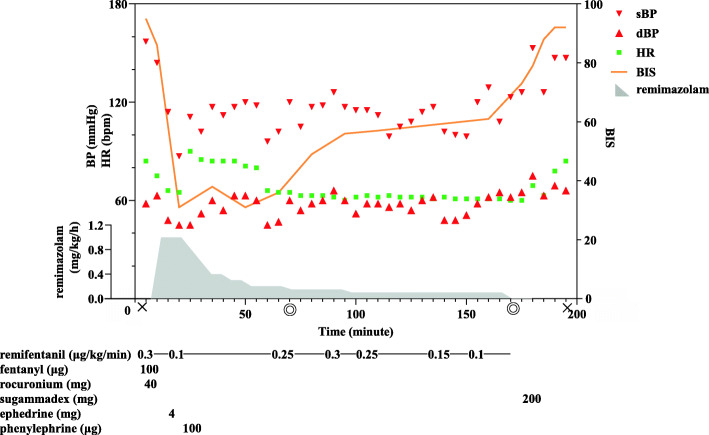
Anesthetic chart for case 2. Double circles and crosses represent the start/end of surgery and start/end of anesthesia, respectively. sBP, systolic blood pressure; dBP, diastolic blood pressure; HR, heart rate; BIS, bispectral index

Anesthetic chart is shown in Fig. 2. In the operating room, she was hemodynamically stable with vital signs as follows: BP, 157/58 mmHg, heart rate, 84 bpm; and oxygen saturation on room air, 94%. BIS value was > 90 before anesthesia. General anesthesia was induced by intravenous administration of fentanyl (100 μg), remifentanil (0.3 μg/kg/min), and remimazolam (1 mg/kg/h). She lost consciousness 3 min after initiation, and subsequently tracheal intubation was performed after administration of rocuronium (40 mg). After the operation was started, the remimazolam dose was adjusted so that the BIS value became 40–60 concomitantly with 0.1–0.3 μg/kg/min of remifentanil infusion. Eventually, the remimazolam dose was decreased to 0.1 mg/kg/h with BIS value of approximately 50–60. The operation was uneventfully completed with a small amount of blood loss in approximately 100 min. During the operation, she remained hemodynamically stable without administration of any vasopressor agents, except when general anesthesia was induced. After completion of the operation, administration of remifentanil and remimazolam was discontinued and 200 mg of sugammadex was administered; 8 min after discontinuation of these agents, she recovered consciousness without flumazenil and received extubation. Any consciousness problem, including resedation or postoperative cognitive function disorder, was not observed during the postoperative course. After rehabilitation, she was discharged on postoperative day 29.

## Discussion

The two patients aged ≥ 95 years had satisfactory intraoperative management with remimazolam. Their hemodynamics were stable during operation, and prompt emergence from anesthesia was achieved. Conversely, it is remarkable that the required maintenance dose could be much lower than the expected dose [2, 3].

Benzodiazepine is widely known to have less circulation effect than other sedatives for general anesthesia, such as propofol or inhalation anesthetics [6, 7]. The effect could be the same for a newly developed benzodiazepine, remimazolam [8]. In the present cases, although our patients were very elderly and relatively hypovolemic, vasopressor agents were almost not required to maintain hemodynamics. This property of remimazolam is beneficial for patients with unstable hemodynamics, including super-elderly patients or patients with hemorrhagic or hypovolemic hypotension, severe cardiac disease, or arteriosclerotic disease. The duration of action of remimazolam is much shorter than that of midazolam, and it appears that remimazolam could be administered without paying too much attention to prolonged effect or resedation after administration of the reversal agent [4]. The extremely short duration of action of remimazolam seems beneficial for a relatively short-term operation. In fact, we could achieve prompt emergence from general anesthesia without the use of reversal agents.

The required dose of remimazolam to maintain BIS value of 40–60 was much lower than those required in previous randomized controlled trials (RCTs) (bolus dose, 6.0 or 12.0 mg/kg/h; mean optimal maintenance dose, 0.99 mg/kg/h for American Society of Anesthesiologists [ASA] class I and II patients and 0.56 mg/kg/h for ASA class III patients) [2, 3]. There is room for examination using appropriate doses, particularly for super-elderly patients. In the present cases, because both patients were very elderly with possible mild hypovolemia, we induced anesthesia with an extremely low dose (bolus dose, 1.2 mg/kg/h for case 1 and 1.0 mg/kg/h for case 2) [3]. However, they lost consciousness only a few minutes after the start of administration. The maintenance doses to achieve BIS values of 40–60 were 0.2 mg/kg/h for case 1 and 0.1 mg/kg/h for case 2, which were much lower than those for ASA class III patients in the previous RCT [3]. Furthermore, in case 1, the maintenance dose of remimazolam was likely to be excessive from the facts that the BIS value was < 40 and electroencephalography often showed burst-suppression patterns [9]. We were hesitant to significantly reduce the dose compared with RCTs, which could be inappropriate. These findings showed that there could be considerable differences in the appropriate dose of remimazolam among individual characteristics, including age and circulatory state as well as other benzodiazepine class anesthetics [10, 11]. In fact, the Japanese phase 2 study shows that both maintenance dose and time to extubation varied widely, and 8% of patients (29/362 cases) need > 30 min to become conscious [2]. The appropriate dose appears to greatly depend on multiple factors, such as age, genetic sensitivity, circulating blood volume, cardiac and liver function, and dose of concomitant opioid. The doses required for our patients were dramatically low; this was primarily owing to their hypersensitivity to sedatives due to their old age, low circulating blood volume, and sufficient dose of remifentanil administration.

It is doubtful that an excessive amount of remimazolam is safe even if the offset period is very short and the reversal agent is available. Deep intraoperative anesthesia is associated with not only postoperative cognitive function disorder but also mortality [12–14]. Most recently, the coronavirus disease 2019 pandemic has resulted in the shortage of sedatives worldwide, due to which clinicians need to ascertain appropriate doses of it [15, 16].

We encountered cases of two patients aged ≥ 95 years who were administered general anesthesia using remimazolam. Remimazolam is a promising agent, particularly for the super-elderly or critically ill patients under general anesthesia to achieve stable hemodynamics. However, dose adjustment needs careful attention. The optimal dose for elderly patients could be much lower as expected. Future research is warranted to clarify the optimal dose for super-elderly patients.

## Data Availability

Not applicable

## Abbreviations

ASA: American Society of Anesthesiologists
BIS: Bispectral index
CKD: Chronic kidney disease
PT: Prothrombin time
RCT: Randomized controlled trials
